# Feasibility of integrating human and animal disease surveillance and reporting in Rwanda: Insights from a mobile reporting pilot and veterinarians' perspectives – a multi-method study

**DOI:** 10.1371/journal.pdig.0000990

**Published:** 2025-08-21

**Authors:** Dieudonne Hakizimana, Janna M. Schurer, Emmanuel Irimaso, Peter Rabinowitz, Joseph Ndagijimana, Janetrix Hellen Amuguni

**Affiliations:** 1 Department of Global Health, University of Washington, Seattle, Washington, United States of America; 2 Department of Global Health Delivery, University of Global Health Equity, Butaro, Rwanda; 3 Center for One Health, University of Global Health Equity, Butaro, Rwanda; 4 Department of Infectious Disease and Global Health, Cummings School of Veterinary, Medicine at Tufts University, North Grafton, Massachusetts, United States of America; 5 College of Agriculture, Animal Science and Veterinary Medicine, University of Rwanda, Nyagatare, Rwanda; 6 Center for One Health Research, Department of Environmental and Occupational Health Sciences, University of Washington, Seattle, Washington, United States of America; 7 Zipline Rwanda (former), One Acre Fund (current), Kigali, Rwanda; North Carolina A&T State University: North Carolina Agricultural and Technical State University, UNITED STATES OF AMERICA

## Abstract

The Rwandan veterinary health system lacks reliable animal disease surveillance data, hindering effective response to zoonotic diseases and other animal health events, including pathogen spillovers with pandemic potential. To address this gap, we piloted a mobile phone reporting system among veterinarians to (1) collect data on animal and human health events and (2) gather insights for future implementations, strengthening the reporting system’s operationalization. A multi-method approach was employed with 14 veterinarians equipped with smartphones. We developed a real-time reporting questionnaire synchronized with a central server and trained the veterinarians to use it during regular field visits. To evaluate the pilot, 11 in-depth interviews were conducted. Quantitative data were analyzed using descriptive statistics, while thematic analysis identified key qualitative themes. Over the study period, veterinarians submitted 1,181 reports through the mobile system, documenting 1,232 cattle disease cases. Common symptoms included inappetence (56.4%) and fever (53.3%). Suspected diseases were primarily East Coast Fever (36.8%) and anaplasmosis (17.4%), with diagnostic tests performed in only 3.6% of cases. Among 3,337 cattle owners, 354 self-reported illness, with 72.6% seeking medical attention. Mobile reporting proved feasible, improving veterinarians’ record-keeping, communication, and collaboration. Key implementation facilitators included training, financial allowances, and technical support, while challenges involved phone capacity and network coverage. Veterinarians leveraged community trust to gather human health data, describing the process as both educational and empowering, and strongly supported the system’s continued use and enhancements. This pilot highlighted the potential of mobile reporting systems to enhance veterinary practice and zoonotic disease surveillance in remote areas. Positive experiences from veterinarians underscore its feasibility, though scaling up requires investments in training, support, incentives, and addressing technological barriers. Future research should evaluate cost-effectiveness and stakeholder readiness to optimize adoption.

## Author summary

Tracking diseases in animals and humans is a challenge in Rwanda. To address this, we tested a mobile phone reporting system for veterinarians to report on animal and human health events. Fourteen veterinarians were trained to use a real-time questionnaire on their smartphones, and 11 interviews were conducted to evaluate the pilot. Veterinarians submitted 1,181 service reports related to the health of cattle and their owners, improving record-keeping and communication. The system was found to be feasible and well-received among veterinary personnel, with the potential to enhance disease monitoring for both animal and human health. Success was attributed to training, allowances, and support, despite challenges related to phone capacity and network coverage. Veterinarians leveraged community trust to gather human health data, extending their role within the community. They strongly advocated for the continued use and enhanced functionality of the system, demonstrating its feasibility and positive experiences during the pilot study. Further improvements and expansion of the system are recommended to strengthen disease surveillance, addressing challenges and building on the identified facilitators.

## Introduction

The disease surveillance system in Rwanda, like those in many African countries, lacks sufficient reliable animal data to effectively respond to endemic and emerging zoonotic diseases. As a result, zoonotic diseases remain a significant public health concern, including highly pathogenic avian influenza (HPAI), Rift Valley fever (RVF), brucellosis, rabies, and viral hemorrhagic fevers such as Marburg virus disease [1,2]. Although national prevalence data are limited, local prevalence data highlight the substantial burden of these diseases. For instance, RVF shows a seroprevalence of 16.8% in cattle, with rates reaching up to 36.9% in some areas [3]. Brucellosis is endemic, with a seroprevalence of 7.4% in cattle, 28.9% in cattle herds, and a 6.1% positivity rate among patients tested in some Rwandan hospitals [4,5]. Limited data on rabies indicate that dog bites accounted for 78% of treated animal bites in 2017 and 2018, though information on rabies-related deaths remains unavailable [6,7]. Data gaps persist for HPAI and viral hemorrhagic fevers, with the recent Marburg virus outbreak highlighting Rwanda’s vulnerability, driven by human-wildlife contact, habitat fragmentation, climate change, and its high-risk regional location [2,8–10].

In recent years, Rwanda has made notable advancements in zoonotic disease surveillance, particularly since the establishment of the national One Health task force in 2013. This initiative promotes collaboration across human, animal, and environmental health sectors [11,12], recognizing that effective zoonotic disease response relies on robust data collection and sharing among these sectors. The integration of Information and Communications Technology (ICT) tools offers significant potential to enhance data collection, streamline communication, and enable seamless sharing, ensuring timely and coordinated action [13]. The human health sector has been using digital systems since 2008, and currently employs the District Health Information Software version 2 (DHIS2) to collect disease data from health facilities and communities on a monthly basis [14–19]. Additionally, the Electronic Integrated Disease Surveillance and Response system (eIDSR), integrated into DHIS2, is used to report cases of epidemic-prone diseases [20,21]. These electronic systems support decision-making and enhance healthcare delivery [14–16,18]. Similarly, the environmental sector uses Climsoft, an open-source software introduced in 2014, to store climatic data and predict disease transmission suitability [22–25].

In contrast, the animal health sector faces significant challenges in data collection and reporting. Community members often self-treat livestock, consulting veterinarians only in severe cases. Even when veterinarians are involved, reporting is hindered by paper-based systems and a complex chain of command. The veterinary system is decentralized to district and sector levels under the Ministry of Local Government rather than the Ministry of Agriculture and Animal Resources [26]. Furthermore, multiple agencies are involved in veterinary services, including the Rwanda Food and Drug Authority (FDA), Ministry of Health (MoH), Rwanda Standards Board (RSB), Rwanda Inspectorate and Competition Authority (RICA), and the Rwanda Environment Management Authority (REMA), resulting in fragmented information flow, inefficient reporting mechanisms, and coordination challenges [27].

Mobile phone reporting systems, which enable real-time data collection and submission, have proven effective in addressing animal health reporting challenges in sub-Saharan Africa [28–32]. For example, Uganda’s Event Mobile Application (EMA-I) significantly improved livestock disease reporting, with the National Animal Disease Diagnostics and Epidemiology Center receiving 126 real-time reports in six months of 2013, compared to 45 and 56 monthly reports submitted through traditional systems in 2012 and 2011 [32]. Similarly, in Tanzania, a mobile phone-based rabies surveillance system generated over 30,000 real-time reports between 2011 and 2016, greatly enhancing disease monitoring [30].

In Rwanda, while mobile phone initiatives exist, their implementation in health care has largely focused on community sensitization rather than addressing disease reporting challenges [33,34]. To operationalize the animal disease reporting system, improve reporting practices, and evaluate the feasibility of collecting both animal and human health data, we developed and piloted a mobile phone reporting system among rural veterinarians. This pilot collected data on cattle diseases and gathered insights to inform future implementations in the veterinary sector. Cattle were prioritized in the pilot for their economic, cultural, and agricultural importance in Rwanda, where they are essential to livelihoods and food security, as highlighted by initiatives like “Girinka” (One Cow per Poor Family), the Rwanda Dairy Competitiveness Program II, and the Rwanda Dairy Development Project, which aim to enhance access to better breeds, improve milk quality, and boost productivity [35]. This manuscript presents the findings from the pilot, including veterinary diagnostic and treatment services reported through the system, human health syndromes reported by cattle owners over the same time period, and veterinarians’ experiences using the mobile reporting system.

## Methods

### Study setting

Rwanda, a low-income and landlocked African country spanning approximately 26.3 square km, had a population of 13.2 million in 2022, estimated to reach 13.8 million in 2024, resulting in one of the highest population densities in Africa at 501 residents per square km [36,37]. The majority, 72.1%, resides in rural areas, with 69% engaged in agriculture. Among households, crop farming is practiced by 63%, and 50% own at least one type of livestock. Cattle (28%), goats (19%), pigs (15%), chickens (12%), and rabbits (6%) are the main types of livestock owned [36].

**Fig 1:**
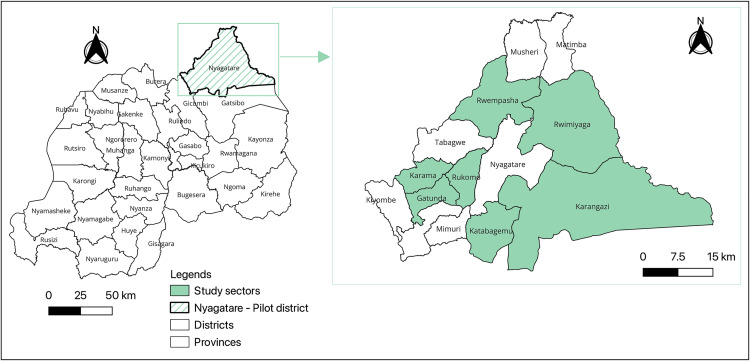
Map of Rwanda displaying the study sectors in Nyagatare District, Eastern Province. The map was created by the author (DH) using QGIS 3.40.1-Bratislava. Its base layer consists of shapefiles sourced from the National Institute of Statistics of Rwanda and publicly available through The World Bank (License: Creative Commons Attribution 4.0): https://datacatalog.worldbank.org/search/dataset/0041453/Rwanda-Admin-Boundaries-and-Villages.

The Gross Domestic Product (GDP) per capita was estimated at 816 USD in 2020, and increased to 1,040 USD in 2023. Agriculture contributed 27% to the GDP, with livestock production accounting for 3%, including contributions from livestock and livestock products [38,39]. Rwanda is divided into five provinces, with each province further divided into districts (Fig 1). Each district is then subdivided into sectors, responsible for implementing development programs, delivering services, and promoting good governance and social welfare. Sectors are further divided into cells, and each cell is comprised of villages. Within this context, the pilot was conducted from June to October 2019 in seven of the 14 sectors in Nyagatare District, Eastern Province to provide critical insights for future interventions (Fig 1) [40]. In Nyagatare, 66.3% of households engage in agriculture, with 59.3% involved in crop farming and 39.2% in livestock husbandry. Regarding livestock, 13.4% of households own cattle, while 21.6% own goats [36].

### Study design

This study employed a multi-method approach. The quantitative component examined veterinary diagnostic and treatment services, as well as human and animal syndromes in farming households. The qualitative component delved into the experiences of veterinarians using the mobile phone-based reporting system.

### The mobile reporting system

To collect veterinary and medical data on cattle in the community and people in farming households, we designed a questionnaire deployed on veterinarians’ smartphones via the Kobo Toolbox application [41,42]. KoBo Toolbox was chosen for its open access, compatibility with tablets and phones, and the ability to function online and offline, facilitating data collection in areas with poor internet connectivity. Survey data is stored on a cloud server and can be downloaded for further analysis [43]. The questionnaire gathered data on communication methods between farmers and veterinarians, clinical examinations of livestock, diagnostic tests conducted by veterinarians, cattle treatment details, and the cost of services. It also included information on human illnesses among individuals in farming households. Developed in English, the questionnaire was pretested by one veterinarian on the study team and further refined during training sessions with participating veterinarians. The first week of implementation served as a pilot phase for veterinarians to familiarize themselves with the reporting system and test the questionnaire; data collected during this period were excluded from the analysis.

The mobile reporting system was piloted in seven of the 14 sectors in Nyagatare District. These sectors were purposively selected to capture data from areas with the highest number of cattle, as reported by the district veterinarian. We enlisted one public and one private veterinarian from each sector. The 14 veterinarians underwent a one-day training to familiarize themselves with the reporting system for documenting services provided in the community.

Veterinarians entered data after providing services in their communities. Typically, they were called to address cattle health problems in a household or farm. They could report data on as many cattle as they treated during a visit but were required to report on at least one cattle treated per visit, along with any human health syndromes observed in the past seven days among members of the same household where the cattle were treated. The information on human health among household members was collected from the head of the household when available. If the head of the household was unavailable, it was collected from another adult (18 years or older).

Veterinarians uploaded data to the Kobo Toolbox server at the end of each day. As a backup, veterinarians were provided with hard copies of questionnaires in case of smartphone issues or power outages in the field. They filled out the data on hard copies and later entered it on a mobile phone, reporting any issues to the study team.

Throughout the implementation pilot, a part-time veterinarian was hired to support field veterinarians by troubleshooting issues and monitoring the reported data for quality assurance. Additionally, each field veterinarian participating in the pilot received $25 per month from the project to cover transportation, communication, internet costs for synchronizing completed questionnaires to the server, and other expenses related to their participation in the pilot.

### Study participants and data collection for pilot evaluation

At the end of the pilot, all participating veterinarians were invited to take part in interviews. An interview guide was used to collect data on their years of experience in the veterinary profession, usual reporting practices, feedback on the training they received for the mobile reporting system, integration of the system into their routine activities, the support provided during the pilot, and their overall experiences with the system, including its benefits and challenges. Additional data were gathered on their experiences requesting human health information from farmers and their recommendations for improving the mobile reporting system for future implementation. To ensure candid responses, an independent data collector conducted the interviews using a structured interview guide. The guide, initially developed in English, was translated into the local language, Kinyarwanda, by the study team. All interviews were conducted in Kinyarwanda and recorded with the veterinarians’ consent.

### Data management and analysis

#### Quantitative data analysis.

Quantitative data were analyzed using frequencies and percentages, calculated in STATA version 14.2 (StataCorp, College Station, Texas). For cattle-related data, we analyzed both visits (defined as a single occasion when a veterinarian provides services at a farm or household) and individual cattle reported (multiple cattle could be treated during a single visit at the same household or farm). For reported visits, we examined the types of veterinary services provided, including the individual contacted, the primary method of contact, and whether payment was made for the services, along with the amount paid. For each treated cattle, we detailed whether a clinical examination (e.g., temperature, respiratory rate, heartbeat rate) was conducted and whether laboratory diagnostic tests were prescribed. We also recorded the types of biological samples collected (e.g., feces, blood, milk, urine), the medicines administered, and their sources. Additionally, we summarized the main symptoms and probable diagnoses reported among the treated cattle.

For human health data, we documented the proportion of people, whose cattle were treated, who consented to provide information on human health issues and illnesses experienced in the past seven days. We recorded the total number of individuals reporting illness during this period and, for those who were sick, detailed their main symptoms, whether they sought medical attention at a health facility, and whether a diagnostic test was performed. If a diagnosis was provided, we included that information as well.

To explore the potential link between human and animal health, we analyzed the presence of gastrointestinal problems, fever, and respiratory issues in three scenarios: both cattle and people in the household, cattle only, or people only. We calculated the odds, along with 95% confidence intervals, of a household with at least one sick cattle also having a sick person for each of these symptoms (fever, gastrointestinal, and respiratory). Additionally, we calculated the positive predictive value (PPV) of sick cattle as a predictor for human illness within the household.

#### Qualitative data analysis.

Qualitative data recordings were transcribed, with identifiable information removed before being submitted to the research team for analysis. A team-based thematic analysis was conducted using Dedoose Version 4.12. The analysis utilized a deductively developed codebook, informed by literature and team expertise, alongside the transcribed data. Additional codes were added inductively during the analysis to capture emerging themes. Two team members experienced in qualitative analysis and fluent in both English and Kinyarwanda conducted the analysis, with one leading and the other verifying and validating the coding. Discrepancies were resolved through discussion. Key themes were identified and described, supported by participant quotes. Selected quotes were translated into English for inclusion in the report.

### Ethical considerations

This study was approved by the Institutional Review Board of the University of Rwanda (Ethical Approval No. 029/19/DRI, dated May 13, 2019). The 14 selected veterinarians provided written informed consent before participating in the study. Verbal consent was obtained from farmers before veterinarians recorded animal and human information in the system.

## Results

### Quantitative results from veterinarians’ reports

#### Veterinary service visits and reporting characteristics.

As presented in Table 1, veterinarians submitted 1,181 visit reports, with 52% (n = 614) from public veterinarians and 48% (n = 567) from private veterinarians. The majority of reports indicated that veterinarians were primarily contacted by the cattle owner or guardian (n = 1,046; 88.6%), with phone communication being the most common method (n = 1,073; 90.9%).

**Table 1:** Summary of veterinary service visits and reporting characteristics during the pilot study (N = 1181).

Variables	Categories		n	%
Type of veterinarian reporting	Public		614	52
	Private		567	48
Who contacted the veterinarian	Relatives		87	7.4
	Scheduled visits		48	4.1
	Owner/Guardian		1046	88.6
Main contact method	By phone		1073	90.9
	Sending someone/ relative person		54	4.6
	Others (Scheduled, visits, etc.)		54	4.6
Medicine administration by the veterinarian per visit	None		75	6.4
	Left the prescription		155	13.1
	Medicine administrated		951	80.5
	Source of administered medicine (n = 951)	The farmer already had them or got from relatives	337	35.4
		The veterinarian already had them	619	65.1
		Bought by the farmer after prescription	33	3.5
The farmer paid for the services	No		678	57.4
	Yes		503	42.6

In terms of treatments, veterinarians administered medications in 80.5% of the reported visits (n = 951) and left prescriptions in 13.1% (n = 155). Among the visits where medications were administered, 65.1% (n = 619) involved medications brought by the veterinarians, while 35.4% (n = 337) involved medications provided by the farmers or obtained from relatives (Table 1).

Regarding payment, veterinarians reported receiving payment in over half of the reported visits (n = 678; 57.4%), while 42.6% (n = 503) reported not receiving any payment. The median cost per treated cattle was 5,000 Rwandan Francs (approximately USD 5.50), with an interquartile range (IQR) of 7,500 Rwandan Francs (approximately USD 8.20), based on the National Bank of Rwanda’s average exchange rate during the pilot period [44] (Table 1).

#### Cattle-related data reported through the system.

Table 2 summarizes data on 1,232 cattle cases treated by veterinarians. Most cattle presented with symptoms, with inappetence (lack of appetite) being the most frequent (56.4%), followed by fever (53.3%), lacrimation (35.6%), cough (31.7%), and swollen lymph nodes (30.4%). Less frequent symptoms included nasal discharge, reduced milk yield, diarrhea, and dry feces. Veterinarians conducted at least one clinical examination in 76.9% of cases, but diagnostic tests were performed in only 3.6% of cases.

**Table 2:** Signs presented by the cattle in Nyagatare District, Rwanda (N = 1,232).

Variable	Category		n	%
Signs observed in cattle	Inappetence		695	56.4
	Fever		657	53.3
	Lacrimation		438	35.6
	Cough		391	31.7
	Swelling of lymph nodes		375	30.4
	Nasal discharge		216	17.5
	Drop in milk yield		215	17.5
	Diarrhea		203	16.5
	Others*		197	16.0
	Dry feces		195	15.8
	Difficulty breathing		145	11.8
	Bloating		90	7.3
	Recumbency or Weakness		90	7.3
	Abortions		88	7.1
	Swollen and warm udder		81	6.6
	Lameness		64	5.2
	Vaginal discharges		49	4
	Stillbirths		48	3.9
	Incoordination		46	3.7
	Weakness		32	2.6
	Death		31	2.5
	Neonatal deaths		26	2.1
Cattle with at least one clinical examination and the variety of examinations conducted among them.	With one clinical examination		948	76.9
	Examinations conducted (n = 948)	Temperature	904	95.4
		Respiratory rate	4	0.4
		Heartbeat rate	2	0.2
Cattle with a diagnostic test	Any diagnostic test		44	3.6
	Diagnostic test performed (n = 44)	Feces	3	6.8
		Blood	27	61.4
		Milk	6	13.6
		Urine	4	9.1

*Other symptoms include those with n < 25, such as lumps, any swelling, preputial discharges, wounds, paralysis, blindness, teeth problems, hair loss, circling, other delivering problems, itching, blood in urine, weight loss/emaciation, etc.

Table 3 displays the probable diagnoses reported by veterinarians. The most common diagnosis was East Coast Fever (theileriosis) (36.8%), followed by anaplasmosis (17.4%). Intestinal worms were suspected in 8% of cases, while mastitis was suspected in 6.4% of cases. Zoonotic diseases, such as brucellosis, were suspected in 6.6%, while others, such as rabies and RVF, were rarely suspected by veterinarians (<0.2%).

**Table 3:** Main probable diagnoses among cattle in Nyagatare District, Rwanda (N = 1,232).

Probable diagnoses	n	%
East Coast Fever (theileriosis)	454	36.8
Anaplasmosis	215	17.4
Intestinal worms	100	8
Brucellosis	83	6.6
Mastitis	82	6.4
Parturition related problems	51	4
Other diagnoses*	44	3.2
Babesiosis	39	3
Traumatic injuries	32	2.3
Trypanosomiasis	30	2.2
Heartwater/Cowdriosis)	27	2
Liver flukes	23	1.5
Foot rot	16	1.1
Milk fever	16	1.1
Lumpy skin disease	14	1
Bovine Ephemeral Fever	12	0.8
Gastrointestinal related problem	10	0.7
Animal bites	9	0.6
Eye related problems	6	0.3
Present of tumor	5	0.3
Black quarter	4	0.2
Infectious Bovine Rhino tracheitis (IBR)	4	0.2
Plant toxicity	4	0.2
Rabies	3	0.1
Rift Valley fever	2	0.1
Bovine Tuberculosis	2	0.1

*Other diagnoses” include a range of conditions reported infrequently by veterinarians. These include: avitaminosis, carbohydrate engorgement, ectoparasite infestation, epistaxis, hernia, hind limb paralysis, hypocalcemia, inflammation of the hoof or udder, suspected rotavirus, mental disorder, navel ill, oedema, pyometra, and unspecified or unknown diseases.

#### Cattle owners’ health-related data reported through the system.

**Fig 2:**
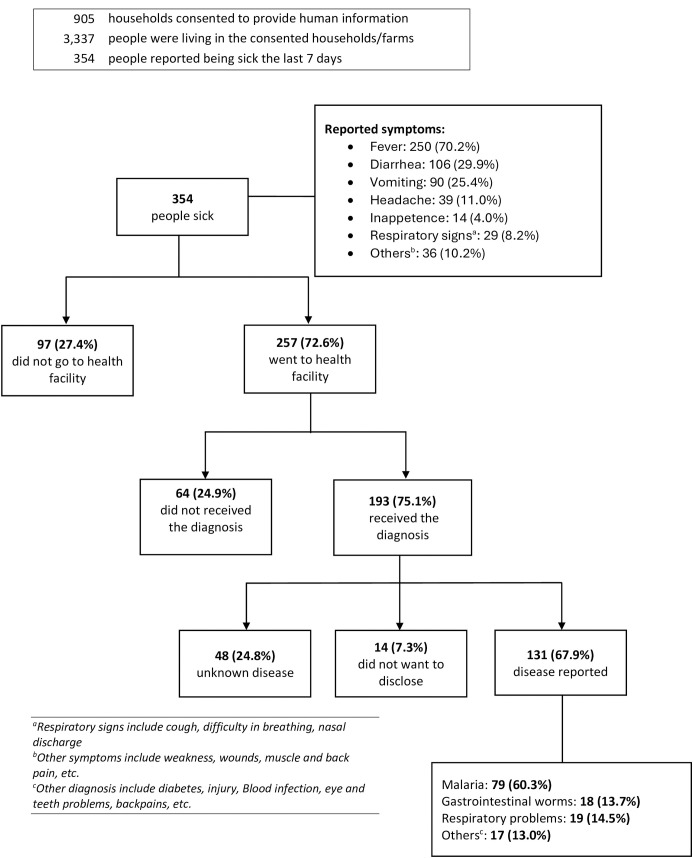
Human illnesses reported in the days prior to veterinary visits.

Regarding human health information, of the 1,181 households visited, 905 (76.6%) agreed to provide data. These households included 3,337 individuals, of whom 354 self-reported being sick in the seven days prior to the veterinarian’s visit. Among those who reported being sick, 70.2% experienced fever, 29.9% had diarrhea, 25.4% experienced vomiting, and 11.0% reported headaches (Fig 2).

Among the 354 individuals who self-reported an illness, 72.6% sought medical attention at a health facility, of which 75% received a diagnosis. Among these, 67.9% were informed of their diagnosis and willingly shared it with the veterinarians, 25% were informed that their disease was unknown, and 7.3% knew their diagnosis but chose not to disclose it. Of the individuals who disclosed their diagnosis, 60.3% reported having malaria, 13.7% reported gastrointestinal worms, and 14.5% reported respiratory problems. Additionally, 13% reported other health conditions, including diabetes, injury, blood infections, eye and dental problems, and back pain (Fig 2).

Table 4 summarizes the analysis of the potential link between human and animal health. Households with at least one cattle experiencing gastrointestinal problems were 0.93 times as likely (95% CI: 0.63–1.39) to have at least one person with gastrointestinal issues, with a PPV of 16.9%. Similarly, households with cattle experiencing fever were 1.27 times as likely (95% CI: 0.92–1.75) to have at least one person with fever, with a PPV of 26.9%. For respiratory problems, households with sick cattle were 1.73 times as likely (95% CI: 0.72–4.26) to have at least one person with respiratory issues, though the PPV was low at 3.6%. When considering any symptom combined, households with cattle exhibiting at least one symptom were 1.42 times as likely (95% CI: 0.95–2.17) to have at least one sick person, with a PPV of 32.6%. None of these odds ratios were statistically significant.

**Table 4:** Potential association between cattle and human health symptoms in households.

			Household with at least one human case sick			OR (95% CI)	PPV
	Symptom		Yes	None	Total		
**Household with at least one animal case sick**	**Gastro-intestinal**	Yes	110	540	650	0.93 (0.63 - 1.39)	16.9%
		None	46	209	255		
		Total	156	749	905		
	**Fever**	Yes	142	385	527	1.27 (0.92 - 1.75)	26.9%
		None	85	293	378		
		Total	227	678	905		
	**Respiratory problems**	Yes	14	373	387	1.73 (0.72 - 4.26)	3.6%
		None	11	507	518		
		Total	25	880	905		
	**At least one of the symptoms**	Yes	245	506	751	1.42 (0.95 - 2.17)	32.6%
		None	39	115	154		
		Total	284	621	905		

CI: Confidence Interval; OR: Odd Ratio; PPV: Positive Predictive Value.

### Results from qualitative analysis: Veterinarians experiences

After analyzing the 11 interviews, five primary themes emerged regarding the veterinarians’ experiences with the mobile reporting system. The system was considered user-friendly, time-efficient, and useful for tracking activities and generating monthly reports. Veterinarians perceived the reporting system as empowering, recognizing their work, and enhancing communication and collaboration among colleagues.

Key facilitators for the system’s implementation included pre-implementation training, provision of allowances to cover costs, and the availability of support. However, technological challenges, such as inadequate network coverage in some areas and limited phone capacity, including compatibility with the system, were significant obstacles. Recommendations for improvement included providing mobile phones for reporting, expanding the system to cover additional veterinary activities beyond cattle disease reporting, and enabling offline and online data entry, picture uploads, and dashboard generation. Table 5 summarizes the themes and their key points.

**Table 5:** Summary of themes and key points from veterinarians’ experiences with the mobile reporting system.

Themes	Key points
Mobile reporting facilitated record-keeping and monthly data aggregation, improving veterinarians’ ability to track their activities.	Easy to use and fit seamlessly into daily routines Improved activity tracking and accurate record-keeping Enabled real-time reporting Streamlined monthly data aggregation, saving time and effort Supported identification of common diseases
Veterinarians perceived mobile reporting as enhancing their services, empowering them, and improving communication and collaboration.	Encouraged more cattle examinations Gave assurance that reports reached decision-makers and were valued Enabled collaborative problem-solving Strengthened peer support and professional communication via WhatsApp
The provision of training, allowances to cover the costs, and support were key factors that facilitated the implementation.	Pre-implementation training helped to use the system effectively Financial support that covered internet, transport, and communication costs Ongoing technical support from the project coordinator Peer support helped troubleshoot technical or app-related issues
The limited capacity of some phones and poor network coverage posed challenges for mobile reporting.	Poor network coverage made it difficult to submit reports in some areas Many phones were incompatible with the system or had limited storage Weak battery life disrupted data entry during fieldwork, requiring use of paper forms
Collecting human health data was facilitated by community trust, serving as education for the community and empowering veterinarians by expanding their role.	Community trust helped veterinarians collect human health data Explaining the purpose of human health questions reduced community hesitation The process helped to educate community members and encouraged care-seeking Veterinarians felt empowered by contributing to both animal and human health Some community members asked health questions beyond veterinarians’ expertise
Veterinarians advocate for continued use and enhanced functionality of the reporting system, including other services, access to data, and report dashboard generation.	Expand the reporting system to cover more services (e.g., vaccination, insemination) and other livestock species Enable access to data across districts and regions Add features like dashboards, data retrieval, and image uploads

#### Mobile reporting facilitated record-keeping and monthly data aggregation, improving veterinarians’ ability to track their activities.

The mobile reporting system was well-liked by veterinarians due to its ease of use and seamless integration into their daily activities. They found it helpful in tracking their activities and keeping accurate records, which was not possible with the previous paper-based system. The system facilitated the aggregation of data for monthly reporting, saving time and effort. Immediate reporting with the system was considered an important feature as it prevented cases from being forgotten, and veterinarians felt confident in aggregating monthly data for reporting. Furthermore, some participants found the records useful in identifying prevalent diseases and treatments in their areas.

*“This mobile report can be used for any veterinary services. It is a good system mainly because you enter data when you are on the field, which is different for the normal reporting we aggregate at the end of the month. You enter the data and send it in real time. Then at the end of the month, you just aggregate, it facilitated us reporting as, it is easy to aggregate data and save time.” (ID 10)*

*“ There was a change because we are now able to keep track of our activities. There was a culture of not keeping a record of our activities, but now [the system] has facilitated this. You can know that in this area, the most prevalent diseases are these because you have the data” (ID 11).*

#### Veterinarians perceived mobile reporting as enhancing their services, empowering them, and improving communication and collaboration.

Some veterinarians noted that since they started reporting, they began asking more questions when treating cattle that they hadn’t previously considered important and conducting additional examinations.

*“Since we started using the mobile reporting system, we have noticed some changes. Firstly, I am now able to respond to anyone who calls me regarding the report. Secondly, during cattle examinations, we have started to examine their eyes and noses because of the reporting requirement, which was not something we were used to doing before (ID2)”*

In addition, they expressed that mobile reporting increased their confidence in reporting, as they viewed it as valid proof for anyone who might want to verify the report. Furthermore, most of them stated that using mobile reporting provided assurance that their reports would reach decision-makers compared to paper-based reporting, which could remain at the local level and leave them uncertain whether it had reached the central level. Some also expressed that when they sent the report, they felt that someone reading it would value their work and make decisions based on the information provided.

*“We support the mobile reporting system for two main reasons. Firstly, it’s an easy-to-use system. Secondly, it reaches many people in a short time. For instance, we can share and discuss reports with other veterinarians on our WhatsApp group, and everyone benefits from experience sharing. In the past, when we gave paper reports, they sometimes stayed in the local office and never reached the intended recipients. However, with mobile reporting, everyone can access and use the reports, which is a significant advantage (ID5)*

*“In my work, I wish that the mobile reporting system could be widely adopted. You can see all these papers; there are so many of them that I wouldn’t have if we were using this system. I hope that the Ministry of Agriculture and Animal Resources, Rwanda Agriculture Board, and other agencies that coordinate us can allocate resources to use this mobile reporting system.” (ID 8)*

Beyond the benefits of the reporting system itself, veterinarians also expressed that the pilot project improved collaboration between them. Some participants mentioned that during the pilot, they were able to take pictures, share them via their WhatsApp group, and collaborate on complicated cases, which helped with communication among all veterinarians. They could assist each other in figuring out appropriate courses of treatment.

*There were many great benefits. Let me start with my colleagues - they are people with specific knowledge. Whenever I encountered a difficult case or disease, I would turn to the group because I knew there were veterinarians with more experience who had seen different cases. I would take pictures of the case and send them, asking for help with a specific case. We would discuss the case amongst ourselves, and some colleagues would recommend which medication to use, or if someone was nearby, they would come and help me. I was able to access that help through this project (ID 1).“*

#### The provision of training, allowances to cover the costs, and support were key factors that facilitated the implementation.

Veterinarians expressed that important facilitators that helped them use the mobile system included the training they received before using it, the availability of support from the project coordinator, whom they could reach out to at any time to address any challenges encountered, receiving an allowance to cover the transportation, communication, and internet costs, and support from their colleagues.

*“During our activities, we received support. Whenever we experienced technical difficulties, we would talk to the [project] coordinator, explain the problem and he would help us solve it (ID 9).”*

*“One great thing about this program is that the internet was readily available. The research team provided it to us every month, and as a result, we did not experience any connectivity issues. While it’s possible for internet connection to be an issue, the research team solved this by providing us with training and money for internet (ID 4).”*

*“I received support from my colleagues in terms of ideas. Sometimes the system would change, or my phone would get stolen, or the application would suddenly disappear from my phone. In those cases, my colleagues would send me the application. Whenever I had difficulty submitting a report, I would ask if they were experiencing similar issues, and they would advise me to try again, or inform me if a specific step had changed (ID 5).”*

#### The limited capacity of some phones and poor network coverage posed challenges for mobile reporting.

Respondents reported experiencing challenges and barriers when using the mobile reporting system. All respondents said that the challenges were mainly technical, as some of the regions where they work have limited internet network coverage. Many participants also reported that the system requirements were incompatible with their phone’s operating system, making it difficult to download the software. Additionally, some participants reported that their phone’s battery would deplete before they could fill in all the information due to the quality of their phones. To overcome these barriers, most veterinarians mentioned carrying a paper collection form or separate notebook to record information when their phone died, and then inputting the information later. As veterinarians were using their personal mobile phones, many expressed a need for proper technology, such as tablets, to collect the information.

*“The barrier I faced is that the system is not available for iPhone. So, I was unable to register the system (ID 8).”*

*“Personally, the region I work in has no network coverage. So, when I was doing field work, it was often impossible for me to fill in the form and submit the report immediately due to network issues. That was the first challenge. The second challenge was phone battery capacity. When we had many cases to input, our phone batteries would deplete before we could enter all the necessary information (ID 06).”*

*“The mobile reporting system is good, but it requires financial capacity and a good telephone. It’s not a lot, but it’s just that it wasn’t organized effectively. If the country needs us to provide full statistics, we should be provided with proper equipment such as telephones, so that no one lacks the equipment as an excuse for not providing reports (ID 4).”*

#### Collecting human health data was facilitated by community trust, serving as education for the community and empowering veterinarians by expanding their role.

Veterinarians reported leveraging their established trust and recognition within the community to effectively gather human health information. They noted that while some community members hesitated due to unfamiliarity with such inquiries, explaining the purpose of the questions helped address concerns and facilitated data collection.

Veterinarians also reported that this process served as an educational opportunity, encouraging care-seeking behavior among community members. They found this dual role empowering, as it expanded their responsibilities beyond animal health and strengthened their relationships with the community. However, they acknowledged occasional challenges when community members posed questions outside their areas of expertise.

*“They were willing to share information, but some were also hesitant. After tending to the cattle, I had to ask about humans, like how many people live in the household. Some were reluctant to respond, and I had to explain the reasons. A few considered it as intruding into personal or private information within their private life (ID2)*

*“It was helpful most of the time; community members often perceive us as caring for animals exclusively, but when we inquire about their health, they quickly realize that we are also health professionals. This not only improves our relationship but also makes them feel cared for. It enhances our collaboration, as they believe we advocate for their needs wherever we go. Asking for human health information was not challenging, and community members appreciated it” (ID3).*

*“Sometimes, they asked questions about their health, and we assured them that we’d help them and do advocacy to address their health problems. There were instances when they asked questions we couldn’t answer, and we told them we’d advocate for them.” (ID4)*

#### Veterinarians advocate for continued use and enhanced functionality of the reporting system, including other services, access to data, and report dashboard generation.

Respondents expressed that there are gaps in the system that need to be addressed to improve its functionality and usability. Many suggested expanding the content to include more diseases, symptoms, and activities such as vaccinations and insemination. Additionally, they also expressed the need for the reporting system to include other livestock species besides cattle.

*“It would be good if this system was expanded… like I said earlier, it is a barrier that the system focuses solely on diseases and treatment activities. The system should include activities on vaccinations, calving, fish and chicken husbandry, and other animals… not just cattle (ID 2).”*

Veterinarians suggested adding features to the system. They expressed the need for the system to generate reports from other districts and sectors so that veterinarians can keep track of the diseases affecting other regions. Additionally, they suggested adding more features such as the ability to access reported data and upload pictures to the reporting system among others. The summary of core and suggested features for an optimal mobile phone reporting system are presented in Table 6.

**Table 6:** Summary of suggested requirements for an optimal mobile phone reporting system.

Category	Core features and suggested improvements
Technology	Real-time reporting Automatic report generation through a dashboard Ability to upload pictures in reporting Ability to enter data offline
Equipment	Provision of phones to veterinarians for using the application Provision of power banks
Support and coordination	Provision of allowances to cover the costs Availability of dedicated staff to troubleshoot technical issues Ability to access reported data Incorporation of feedback mechanisms for continuous improvement
Contents	Expansion of the reporting content to include other activities such as inseminations Revision of the flow of the questions for improved clarity and efficiency

*“I would suggest that the system be expanded beyond treatment. Secondly, there should be a way to connect so that diseases we are reporting… you see those [who report diseases] of Nyagatare report on Nyagatare and Rwempasha… there should be a way to see them so that I can understand what their situation is like (ID 2).”*

## Discussion

This study highlights the transformative potential of mobile reporting systems in enhancing veterinary practice, particularly in resource-limited settings. By improving reporting, fostering collaboration among veterinarians, and integrating human health data collection, the system strengthens zoonotic disease surveillance and advances a One Health approach. Additionally, the study identified symptomatic diagnoses and limited use of diagnostic tests in cattle health, presenting clear opportunities to enhance surveillance. Positive experiences reported by pilot veterinarians, coupled with their recommendations to scale up the system, underscore its feasibility. However, for successful implementation and scale-up, strategic investments are needed in quality training, ongoing support, and incentivization strategies. Addressing technological barriers, such as providing standardized offline-capable devices and improving network coverage, is equally critical. Furthermore, enhancing the system with additional features, evaluating their cost-effectiveness, and considering stakeholder readiness and perspectives will be key to optimizing adoption and maximizing the system’s impact.

### Contribution to One Health and zoonotic disease surveillance

The results highlight the potential of mobile reporting systems to integrate human health data collection, advancing a One Health approach. By leveraging their trusted roles, veterinarians collected human health information during animal health visits, demonstrating a practical method to strengthen zoonotic disease surveillance, particularly in rural areas with limited surveillance. This integration highlights a promising avenue for bridging gaps in rural health systems where dedicated human health surveillance mechanisms are often lacking [45].

However, these dual responsibilities require careful management to prevent overburdening veterinarians and ensure their continued focus on core veterinary duties. Policymakers should explore incentivization strategies to balance workloads, maintain motivation, and support veterinarians in fulfilling these expanded roles effectively. Additionally, equipping veterinarians with the necessary skills through targeted training in human health topics, delivered via online modules or in-service programs, is essential for building capacity and ensuring the quality of health data collected during animal health visits [46,47]. These strategies are critical to sustaining motivation and ensuring veterinarians contribute meaningfully to public health initiatives without compromising the quality of veterinary care.

Our findings further highlight that households and farms often exhibit similar syndromes in humans and animals, such as gastrointestinal issues and fever—common signs of zoonotic diseases. However, no significant association was found between symptoms in cattle and humans, likely due to the study’s focus on short-term symptom observation and the non-exclusive nature of these symptoms. Despite low predictive values, these findings underscore the valuable role of surveillance systems in assessing the health impacts of human-animal cohabitation. Future research should explore more robust methods of linking human and animal health data, such as laboratory testing and antimicrobial resistance studies, to deepen our understanding of zoonotic disease transmission.

Integrating human health considerations into veterinary visits provides a valuable opportunity to advance the One Health approach and address broader public health challenges, including antimicrobial resistance. In this study, over 30% of household medications were obtained informally, and 27% of individuals reported not seeking care at a health facility when sick. These practices pose significant safety risks and contribute to the growing issue of antimicrobial resistance [48,49]. Veterinarians can play a crucial role in mitigating these challenges by educating communities, encouraging timely care-seeking behaviors, promoting the safe use of medications, and supporting public health initiatives.

### Enhancing veterinary service quality and collaboration diagnostic gaps

The adoption of mobile reporting systems not only improved data submission but also enhanced collaboration among veterinarians. The system encouraged more thorough examinations, such as inspecting cattle eyes and noses, which were not routine practices before the pilot. Additionally, veterinarians reported improved communication, as the system prompted them to seek support for complex cases, fostering a culture of collaboration. These shifts can contribute to greater consistency in diagnostics and treatment, thereby improving the overall quality of veterinary services. Policymakers should consider scaling mobile reporting systems and integrating them into national veterinary guidelines to institutionalize best practices, ensuring that livestock receive high-quality care across regions.

This study also identified critical gaps in diagnostic capabilities. Veterinarians relied predominantly on symptomatic diagnoses, which are insufficient for confirming diseases requiring laboratory testing. This limitation likely explains the underreporting of zoonotic diseases such as brucellosis and RVF, despite their public health significance [3–5]. To address these gaps, investments in affordable diagnostic tools, such as rapid tests and point-of-care diagnostics, are crucial. Strengthening laboratory capacity and integrating diagnostic services into mobile reporting systems would improve disease confirmation and surveillance outcomes [45,50].

### Facilitators and barriers to implementation and potential for system enhancements

Key facilitators of successful implementation included training, financial assistance, and ongoing technical support. These efforts equipped veterinarians with the skills, resources, and motivation to adopt the system effectively, aligning with evidence from prior studies emphasizing the importance of capacity-building for digital health interventions [51,52]. Importantly, financial allowances played a significant role in maintaining user engagement. As highlighted in similar contexts, the absence of incentives can demotivate participation, underscoring the need for sustainable funding models [53]. Policymakers must prioritize these facilitators as integral components of implementation strategies, ensuring that mobile reporting systems are not only adopted but also sustained over time.

However, veterinarians faced significant technological barriers, including phone compatibility issues, inconsistent network coverage, and a lack of standardized equipment. These challenges were particularly pronounced in rural areas, where telecommunication infrastructure remains underdeveloped [54,55]. Many veterinarians reported difficulties submitting data due to unreliable network access, emphasizing the critical need for offline functionality to ensure uninterrupted operation in low-connectivity settings. Providing program-specific devices tailored to system requirements, coupled with robust offline capabilities, would enhance usability and reliability. Additionally, bridging telecommunication gaps through infrastructure development is essential to extend the benefits of digital veterinary services to underserved areas.

Veterinarians proposed several enhancements to the system, including picture uploads, automated dashboards, and expanded content to cover additional livestock species and veterinary activities. These improvements could streamline reporting processes, enhance data utility, and support evidence-based decision-making. However, these advancements must be weighed against cost implications, which remain a key barrier to scaling mobile health systems [56,57]. While this study did not conduct a cost-effectiveness analysis, future research should explore the financial feasibility of such enhancements and identify collaborative funding mechanisms involving public and private stakeholders.

Finally, while this pilot demonstrated the potential of mobile reporting systems to enhance data quality and foster collaboration, scaling these systems requires comprehensive integration into broader national strategies and alignment with veterinary and public health priorities. Collaborative partnerships among governments, private sectors, and development agencies will be crucial to providing the necessary resources, technical expertise, and policy support. Addressing the identified challenges and applying lessons from this pilot can transform veterinary practice and enhance public health outcomes in resource-limited settings.

### Strengths and limitations

This study provides valuable insights into veterinary services, focusing on symptoms, diagnostic practices, and challenges in implementing mobile reporting systems. By integrating qualitative methods and veterinarians’ real-world experiences, it highlights opportunities to enhance disease management and veterinary practices. Notably, this was one of the first studies, to our knowledge, to combine animal and human health data collection through veterinarians, demonstrating the potential of a One Health approach to address interconnected health challenges. These findings provide a strong foundation for scaling mobile reporting systems in animal health, significantly advancing the field of digital veterinary systems.

Despite its contributions, the study has some limitations. The purposive selection of a single district, specific sectors, and veterinarians ensured relevance to the local context while making the most of limited funding, though it may have limited generalizability. Voluntary reporting provided practical insights but depended on veterinarians’ willingness to participate. Allowances may have influenced positive feedback but highlight the role of incentives in under-resourced settings.

Additionally, the study did not include perspectives from policymakers or community members, nor did it compare mobile reporting with existing systems. These omissions align with its focus on veterinarians as primary users, a critical first step in evaluating feasibility but also veterinarians, especially in private practice, rarely report diseases routinely, comparisons with underutilized systems would provide limited value. Future research should address these gaps by assessing cost-effectiveness, incorporating broader stakeholder views, and evaluating Rwanda’s readiness to adopt mobile reporting systems.
